# Phase I dose-finding study of eribulin and capecitabine for metastatic breast cancer: JBCRG-18 cape study

**DOI:** 10.1007/s12282-017-0798-4

**Published:** 2017-08-31

**Authors:** Masaya Hattori, Hiroshi Ishiguro, Norikazu Masuda, Akiyo Yoshimura, Shoichiro Ohtani, Hiroyuki Yasojima, Satoshi Morita, Shinji Ohno, Hiroji Iwata

**Affiliations:** 1 Department of Breast Oncology, Aichi Cancer Center, 1-1 Kanokoden, Chikusa-ku, Nagoya, 464-8681 Japan; 2 Department of Target Therapy Oncology, Graduate School of Medicine, Kyoto University, Kyoto, 606-8501 Japan; 3 Department of Surgery, Breast Oncology, NHO Osaka National Hospital, Osaka, 540-0006 Japan; 4 Division of Breast Surgery, Hiroshima City Hiroshima Citizens Hospital, Hiroshima, 730-8518 Japan; 5 Department of Biomedical Statistics and Bioinformatics, Graduate School of Medicine, Kyoto University, Kyoto, 606-8501 Japan; 6 Breast Oncology Center, Cancer Institute Hospital of JFCR, Tokyo, 135-8550 Japan

**Keywords:** Breast cancer, Capecitabine, Eribulin, Metastases, Pharmacokinetics

## Abstract

**Background:**

Eribulin is a nontaxane microtubule inhibitor with activity in patients with metastatic breast cancer (MBC). We conducted a phase I dose-finding study of eribulin and capecitabine in patients with MBC pretreated with anthracycline and taxane.

**Methods:**

Women with MBC aged ≤70 years were enrolled. A 3 + 3 dose escalation design was used: level 0 dosing, eribulin (1.4 mg/m^2^ intravenously on days 1 and 8) plus capecitabine [825 mg/m^2^ orally twice daily (BID)]; 2-weeks-on, 1-week-off in a 21-day cycle. If there were no dose-limiting toxicities (DLTs), level 1 capecitabine dose was 1000 mg/m^2^ BID. The primary objective was to determine maximum tolerated dose, DLTs, and recommended dose (RD). Secondary objectives included pharmacokinetics, safety, and best overall response rate.

**Results:**

Nine women with MBC were enrolled; six at level 0, three at level 1. One patient had grade 4 DLTs at level 0 (serum creatinine 7.65 mg/dL and uric acid 13.4 mg/dL), considered associated with study drugs. Level 1 dosing was taken as the RD. Neutropenia was the most common ≥grade 3 toxicity. Pharmacokinetic parameters of eribulin were not influenced by co-administration of capecitabine. Of three patients in level 1, one achieved partial response and one had prolonged stable disease.

**Conclusion:**

Eribulin with capecitabine in the level 1 dosing schedule was associated with manageable toxicities and promising clinical activity. This combination is recommended for phase II investigation.

**Electronic supplementary material:**

The online version of this article (doi:10.1007/s12282-017-0798-4) contains supplementary material, which is available to authorized users.

## Introduction

According to estimates by the World Health Organization, breast cancer is the second most common cancer in the world and by far the most frequent cancer among women, with 1.67 million new cancer cases diagnosed in 2012 (25% of all cancers) [1]. Despite improvements in the strategies targeting the primary tumour, metastatic disease remains the most common cause of death in those patients with breast cancer who do not survive, and hence poses a major therapeutic challenge [2]. As metastatic breast cancer (MBC) is currently incurable, the goals of therapy are to prolong survival, palliate symptoms, and improve quality of life [3]. Anthracycline- or taxane-based regimens are often chosen for treatment. A limited number of agents are currently available for treatment of patients with MBC who have been pretreated with anthracycline and taxane and there is an urgent need to develop novel treatments for these patients.

Eribulin, a nontaxane microtubule dynamics inhibitor belonging to the halichondrin class of antineoplastic agents has a mechanism of action distinct from currently available taxanes [4, 5]. It binds at microtubule ends to a single site on tubulin to suppress dynamic instability, unlike taxanes. Eribulin has received U.S. Food and Drug Administration and European Medicines Agency approval for the treatment of locally advanced or MBC refractory to both anthracyclines and taxanes [6]; additionally, it gained approval in Japan in May 2010 [7]. Eribulin showed a significant and clinically meaningful improvement in overall survival (OS) compared to treatment of physician’s choice in patients with heavily pretreated MBC in a phase III study [8].

Capecitabine has the unique ability to convert into 5-fluorouracil (5-FU) by the enzyme thymidine phosphorylase, which is highly active in tumours [9]. 5-FU exerts its cytotoxic effect by inhibiting DNA, RNA, and protein synthesis and has been studied in various clinical trials in combination with other cytotoxic agents [6, 10]. Patients with locally advanced or MBC previously treated with an anthracycline and a taxane have shown marked (and similar) improvement in functioning assessed by health-related quality of life measures following treatment by eribulin or capecitabine [11].

Although a number of combination regimens have been reported for the treatment of patients with MBC, only a few demonstrated superior survival benefits compared with single-agent use. To date, docetaxel plus capecitabine [12] and gemcitabine plus paclitaxel [13] have demonstrated superior survival benefit in patients with MBC. Currently, eribulin-based combinations are being actively studied and attracting attention from oncologists engaged in particularly difficult-to-treat settings such as triple-negative breast cancer (TNBC) [7].

A phase I dose-escalation study of eribulin in combination with S-1, an oral fluoropyrimidine capsule formulation that consists of a prodrug of 5-FU, demonstrated that the pharmacokinetic profiles of each drug appeared to be unaffected by co-administration and a promising antitumour activity for the combination therapy [14]. However, the authors recommended the intermediate dose level for phase II due to occurrence of febrile neutropenia in three out of six patients at level 3 dosing [14]. Another oral fluoropyrimidine capsule formulation of a prodrug of 5-FU, capecitabine, may also represent a potentially new combination with eribulin for the treatment of patients with MBC. The key toxicities of eribulin and capecitabine do not overlap [11]. Combination therapy of eribulin and capecitabine was expected to have a synergistic effect on antitumour activity, with manageable toxicity [15–17]. Given the potential impact of race/ethnicity and cultural differences on drug disposition, efficacy, and toxicity, we conducted a phase I dose-escalation study to evaluate the safety and pharmacokinetic profiles of combination use of eribulin and capecitabine in Japanese patients with MBC. Furthermore, we determined a recommended dose (RD) for the phase II study.

## Patients and methods

### Patients

Women with MBC (i.e. metastatic, inoperable, recurrent, or locally advanced breast cancer) who had been previously treated with anthracycline- or taxane-based regimens were eligible for this study. Major inclusion criteria included age ≤70 years, Eastern Cooperative Oncology Group performance status of 0–1, haematologic or nonhaematologic adverse events ≤grade 1, and life expectancy ≥6 months. Additional eligibility criteria included adequate bone marrow, hepatic, and renal function, as defined by laboratory values including neutrophil count ≥2000/mm^3^, platelet count ≥100,000/mm^3^, haemoglobin ≥9.0 g/dL, total bilirubin ≤2.0 mg/dL, aspartate aminotransferase and alanine aminotransferase ≤3.0 times the upper limit of normal, and serum creatinine ≤1.5 mg/dL. Presence of a target lesion was not mandatory for a patient to be enrolled in this study.

Major exclusion criteria included administration of capecitabine immediately before study entry, severe allergic reaction to any of the study drugs, serious complications, active brain metastasis, and pregnancy. Patients who were considered ineligible by the investigator were also excluded.

### Study design

This was a phase I, multicentre, open-label, dose-escalation study of eribulin in combination with capecitabine. Patients were recruited from five medical centres. To find the maximum tolerated dose (MTD), a standard 3 + 3 dose escalation design was used.

Eribulin was administered intravenously on days 1 and 8 and capecitabine orally twice daily (BID) in a 2-weeks-on and 1-week-off schedule, in a 21-day cycle. The level 0 dosing was eribulin 1.4 mg/m^2^ and capecitabine 825 mg/m^2^ BID. If no patient experienced a dose-limiting toxicity (DLT), the dose was escalated to level 1 (eribulin 1.4 mg/m^2^ + capecitabine 1000 mg/m^2^ BID) in subsequent patients. If no DLT was experienced at this level, level 1 would be recommended for the phase II study. If neither level 0 nor level 1 were tolerable, patients would enter a lower level. At level −1, the dose schedule was eribulin 1.4 mg/m^2^ + capecitabine 600 mg/m^2^ BID and for level −2, the dose was eribulin 1.1 mg/m^2^ + capecitabine 600 mg/m^2^ BID. If two or more of six patients at level −2 experienced DLT(s), the recommended dose of eribulin and capecitabine in combination use for the phase II study would not be decided.

In the first cycle, the initial dose of eribulin administration was set as day 1 and the initial dose of capecitabine was orally administered the day before day 1 (day 0). The administration of eribulin was delayed if a patient was reported to have specified changes in haematologic or blood chemistry parameters, i.e. neutrophil count <500/mm^3^, platelet count <50,000/mm^3^, haemoglobin <9.0 g/dL, total bilirubin >2.0 mg/dL, aspartate aminotransferase and alanine aminotransferase >3.0 times the upper limit of normal, or serum creatinine >1.5 mg/dL. The administration of capecitabine was interrupted if a patient had grade ≥2 hand–foot syndrome. Any change in the schedule of study drug administration was based on the physician’s assessment of the patient’s condition.

In the second cycle, the administration of eribulin was delayed if a patient had neutrophil count <1500/mm^3^, platelet count <100,000/mm^3^, haemoglobin <9.0 g/dL, total bilirubin >2.0 mg/dL, aspartate aminotransferase and alanine aminotransferase >3.0 times the upper limit of normal, or serum creatinine >1.5 mg/dL. The administration of study drugs continued until progression of disease or intolerable or unmanageable toxicities. Concomitant use of other drugs for supportive care was permitted; however, prophylaxis for neutropenia with granulocyte colony-stimulating factor was not permitted. Other anticancer therapies were not permitted.

The study protocol was approved by local institutional review boards and ethics committees at each study site. This study was conducted in accordance with the Good Clinical Practice guidelines, the Japanese Guidelines for Clinical Research of the Ministry of Health, Labour and Welfare, and the Declaration of Helsinki, as well as other applicable regulatory requirements. All participants provided written informed consent prior to the study entry. The present study has been registered with the University Hospital Medical Information Network (UMIN) Center (ID: UMIN 000009611).

### Assessment

The primary objectives were to determine DLTs and MTD, as well as the RD for the subsequent phase II study. Secondary objectives included the determination of pharmacokinetics, safety, preliminary assessment of antitumour activity such as best overall response rate, progression-free survival (PFS; the time from the initiation of the combination therapy with eribulin and capecitabine until exacerbation of the primary disease), and OS (the time from initiation of therapy until death from any cause). Discontinuation of therapy was defined as the last date of protocol treatment or last observation date of patients who discontinued treatment for reasons other than exacerbation of primary disease.

The DLT and MTD were evaluated in cycle 1. The MTD was indicated by the incidence of DLT(s) in two or more of the patients enrolled in level 0. DLTs and other adverse events (AEs) were assessed according to National Cancer Institute Common Terminology Criteria for Adverse Events (NCI CTC-AE) version 4.0. DLTs were defined as ≥grade 3 neutropenia with fever requiring intravenous administration of antibiotics, ≥grade 3 neutropenia with bacteraemia or sepsis, grade 4 thrombocytopenia, any ≥grade 3 nonhaematologic AEs, and any AEs leading to a delay of >2 weeks in study drug administration.

For assessment of pharmacokinetics, blood samples were taken during cycle 1. The pharmacokinetic parameters of eribulin, capecitabine, and capecitabine metabolites [5′-deoxy-5-fluorocytidine (5′-DFCR), 5′-deoxy-5-fluorouridine (5′-DFUR), and 5-FU] were assessed. The plasma concentration of eribulin was evaluated at baseline, within 1 min after administration, and 0.5, 1, 2, 4, 6, and 168 h after the administration of eribulin on day 1 of cycle 1. The plasma concentrations of capecitabine and capecitabine metabolites were evaluated 1, 2, 4, and 6 h after administration of capecitabine on day 0 and on day 1 in cycle 1. Plasma concentration versus time data were fitted to non-compartmental analysis using WinNonlin version 6.3 (Certara LP, Princeton, NJ, USA). The SAS system 9.4 (SAS Institute Inc, Cary, NC, USA) was used for statistical analysis. *P* < 0.05 was considered statistically significant.

For assessment of safety, AEs were recorded and graded according to CTC-AE version 4.0. For preliminary assessment of efficacy, best overall tumour response and disease progression were measured based using the Response Evaluation Criteria in Solid Tumors version 1.1. Baseline target lesions were evaluated within 14 days before the first administration of the study drugs.

## Results

### Patients

Nine women with MBC were enrolled in this study between December 2012 and December 2013. Patient characteristics including histological evaluation are summarised in Table 1. All patients had been previously treated with anthracycline- or taxane-based regimens in adjuvant settings or as treatment for MBC.

**Table 1:** Patient demographics and baseline characteristics

	Total	Level 0	Level 1
	*N* = 9	*N* = 6	*N* = 3
Mean age (year)	47.1 (34–57)	46.0 (34–57)	49.3 (36–63)
ECOG PS, *n*			
0	8	6	2
1	1	0	1
Histology, *n*			
ER (+) and/or PgR (+)	8	5	3
ER (−) and PgR (−)	1	1	0
HER2 (+)	0	0	0
Metastasis sites, *n*	9	6	3
Bone	7	5	2
Lung	3	2	1
Liver	8	5	3
Soft tissue	2	1	1
Evaluable target lesion, *n*	7	4	3
Breast cancer surgery, *n*	8	5	3
Chemotherapy			
Anthracycline for adjuvant	7	4	3
Taxane for adjuvant	5	3	2
Anthracycline for MBC	2	2	0
Taxane for MBC	5	3	2
Number of chemotherapy for MBC			
0	2	1	1
1	3	2	1
2	1	1	0
3 or more	3	2	1

*ECOG PS* Eastern Cooperative Oncology Group performance status, *ER* estrogen receptor, *HER2* human epidermal growth factor receptor 2, *PgR* progesterone receptor, *MBC* metastatic breast cancer

### DLTs and dose level changes

Among the initially enrolled three patients for level 0, one patient experienced DLTs. At the time when this patient experienced the DLT, the patient had normal serum potassium and calcium levels but showed elevated serum phosphate level (4.8 mg/dL) along with grade 4 increase in serum creatinine level (7.65 mg/dL) and grade 4 hyperuricaemia (13.4 mg/dL); both events occurred on day 14 of cycle 1, and were considered to be associated with the study drugs. In view of the DLTs, an additional three patients received level 0 dosing, none of whom experienced any DLTs. Therefore, three patients were enrolled for level 1 and none experienced DLTs. Thus, a dosing schedule of eribulin at 1.4 mg/m^2^ on days 1 and 8 combined with capecitabine 1000 mg/m^2^ BID in a 2-weeks-on and 1-week-off schedule in a 21-day cycle was tolerable and chosen for further investigation in the phase II study. Because no further dose escalation was performed, the MTD for this drug combination was not evaluated.

### Safety and tolerability

**Fig. 1:**
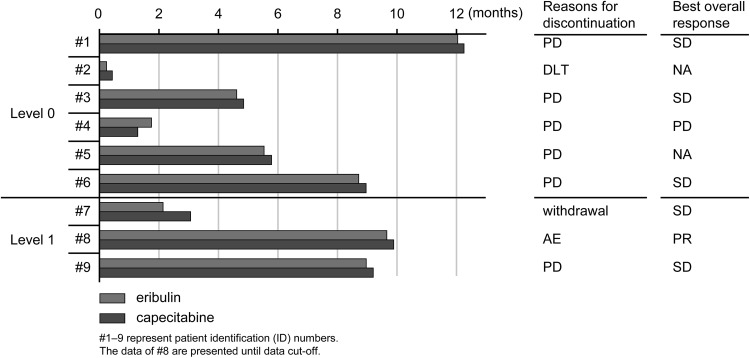
Duration of administration of the study drugs (*N* = 9). *AE* adverse event, *DLT* dose-limiting toxicity, *NA* not available, *PD* progressive disease, *PR* partial response, *SD* stable disease

At the data cutoff on 5 March 2015, eight patients (six at level 0 and two at level 1) had discontinued the study (Fig. 1). Five patients at level 0 discontinued due to progressive disease, one each at level 0 and level 1 due to AEs, and one at level 1 due to study withdrawal. Overall, the patients received 7.5 and 9.3 cycles of eribulin and capecitabine at level 0 and level 1, respectively.

An overview of AEs is shown in Table 2. The most common grade 3 or 4 toxicity was neutropenia. Among the six patients who received the level 0 dosing schedule, grade 3 neutropenia was observed in one, and grade 4 neutropenia in five of them, while grade 3 neutropenia was observed in one patient and grade 4 neutropenia in two at level 1. However, febrile neutropenia was not observed at either of the two levels. Among nonhaematologic toxicities, grade 4 increases in serum creatinine and uric acid were observed in one patient and were considered to be DLTs. The occurrence of these events was possibly attributable to tumour lysis syndrome. The other major grade 1 or 2 nonhaematologic toxicities were elevated serum concentrations of aspartate aminotransferase and alanine aminotransferase, constipation, peripheral neuropathy, and fatigue. There were no treatment-related deaths. Treatment delay occurred in five patients; four patients with neutropenia and one patient in whom delay was not related to any AE. Treatment withdrawal was reported in two patients with peripheral neuropathy.

**Table 2:** Adverse events

	Level 0 (*N* = 6)				Level 1 (*N* = 3)			
	Grade 1	Grade 2	Grade 3	Grade 4	Grade 1	Grade 2	Grade 3	Grade 4
Neutropenia	0	0	1	5	0	0	1	2
Thrombocytopenia	0	0	0	0	0	0	0	0
Anaemia	2	1	0	0	1	0	0	0
Febrile neutropenia	0	0	0	0	0	0	0	0
Hand–foot syndrome	1	0	0	0	1	0	0	0
Constipation	2	1	0	0	0	0	0	0
Peripheral neuropathy	2	0	0	0	1	0	0	0
Fatigue	1	0	0	0	1	0	0	0
Oral mucositis	2	0	0	0	0	0	0	0
Muscle pain	2	0	0	0	1	0	0	0
Serum creatinine increase	0	0	0	1	0	0	0	0
Uric acid increase	0	0	0	1	0	0	0	0
Alanine aminotransferase increase	6	0	0	0	1	0	0	0
Aspartate aminotransferase increase	5	0	0	0	1	0	0	0

### Pharmacokinetics

**Fig. 2:**
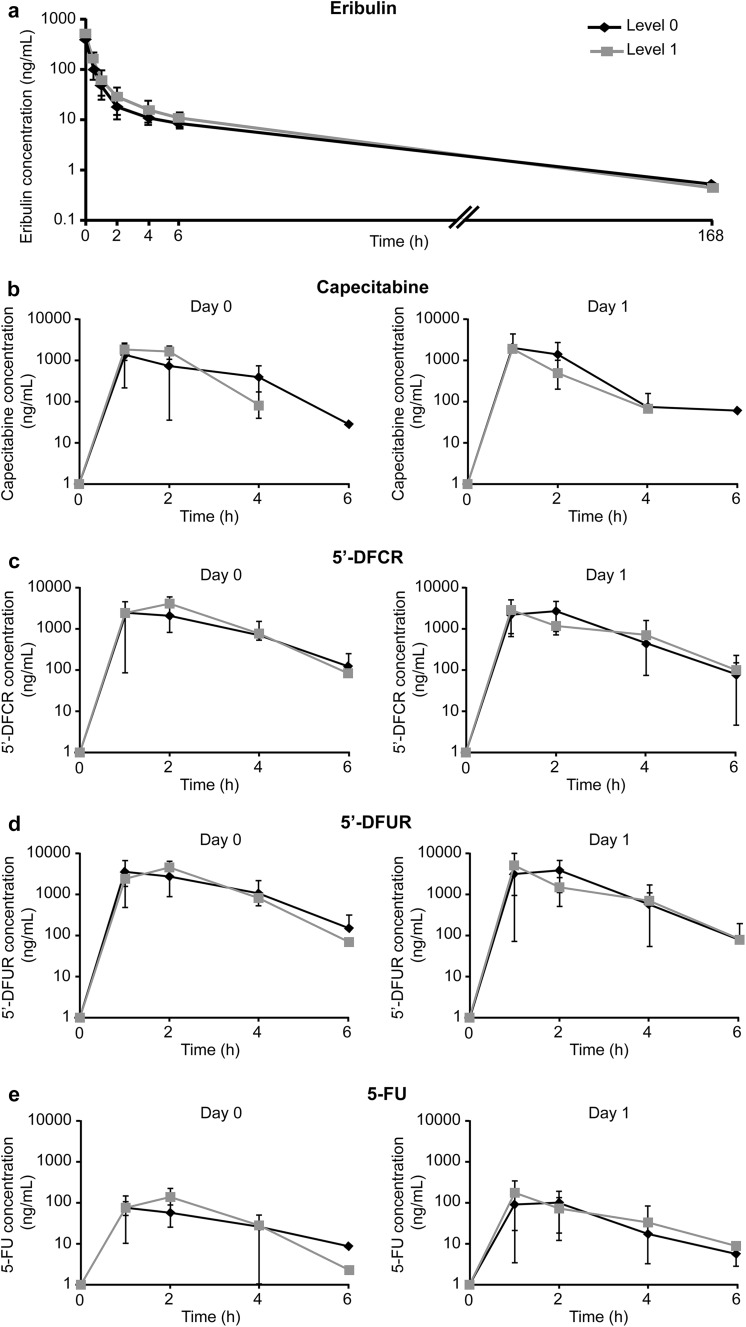
The time-course changes in the plasma concentration of eribulin, capecitabine, and capecitabine metabolites. **a** Eribulin. The first time plot was 1 min after the administration of eribulin. **b** Capecitabine. **c** 5′-DFCR. **d** 5′-DFUR. **e** 5-FU

All nine patients in this study were included in the pharmacokinetic analysis. The time-course changes in the plasma concentrations of eribulin, capecitabine, and capecitabine metabolites are presented in Fig. 2a, b. The baseline concentration of eribulin was below the lower limit of quantification. Plasma concentration of eribulin peaked at 1 min after the infusion, and declined rapidly. The distribution profile for eribulin was multiphasic, with a rapid alpha (tissue redistribution) phase followed by a prolonged beta (elimination) phase. Although level 1 dosage resulted in a higher maximum concentration (*C*_max_) and area under the curve (AUC) for eribulin, the prolonged elimination phase of the escalated dose was similar to that of the level 0 dose. Therefore, increase in capecitabine dosage (level 1 dosing) did not affect the elimination of eribulin. Capecitabine was also rapidly metabolised into 5′-DFCR, 5′-DFUR, and 5-FU, as indicated by the early *C*_max_ achieved by these metabolites (Fig. 2c–e). The *C*_max_ and AUC for capecitabine and its metabolites are similar between day 0 and day 1 (after eribulin administration). Therefore, combination use of eribulin did not affect the pharmacokinetic profile of capecitabine and its metabolites. Other pharmacokinetic parameters of eribulin and capecitabine are summarised in Table 3 and Supplementary Table 1. The differences between the plasma concentrations of eribulin, capecitabine, and capecitabine metabolites at the two doses were similar. The pharmacokinetic parameter estimates for eribulin and capecitabine in the present study were consistent with those observed with eribulin monotherapy [18] and capecitabine monotherapy [19].

**Table 3:** Pharmacokinetic parameters of eribulin

Parameter	Level 0						Level 1					
	Eribulin		Capecitabine				Eribulin		Capecitabine			
	Day 1		Day 0		Day 1		Day 1		Day 0		Day 1	
	*n*	mean (SD)	*n*	mean (SD)	*n*	mean (SD)	*n*	mean (SD)	*n*	mean (SD)	*n*	mean (SD)
*C*_max_ (µg/mL)	6	0.391 (0.132)	6	1.687 (0.896)	3	2.898 (1.880)	3	0.521 (0.066)	6	2.006 (0.780)	3	2.212 (2.420)
AUC_0–t_ (h µg/mL)	6	0.982 (0.189)	6	2.814 (0.707)	3	4.470 (2.760)	3	1.233 (0.376)	6	4.426 (1.151)	3	2.713 (2.542)
t_1/2_ (h)	5	38.1 (7.3)	6	0.41 (0.08)	3	0.44 (0.19)	1	34.6	6	0.48 (0.27)	3	0.34 (0.13)
CL (L/h/m^2^)	5	1.4 (0.3)	6	368.8 (213.7)	3	850.7 (1549.4)	1	1.2	6	318.0 (111.2)	3	407.0 (377.3)
Vz (L/m^2^)	5	77.7 (18.7)	6	233.0 (155.2)	3	539.9 (921.4)	1	61.9	6	249.6 (222.2)	3	243.2 (287.3)
MRT (h)	5	17.9 (8.5)	6	1.3 (1.2)	3	1.0 (0.8)	1	14	6	1.4 (0.3)	3	0.7 (1.0)

*AUC*_*0–t*_ area under the plasma concentration–time from 0 to 168 h for eribulin and from 0 to 6 h for capecitabine, *C*_*max*_ maximum plasma concentration, *CL* clearance, *MRT* mean residence time, *SD* standard deviation, *t*_*1/2*_ half-life, *Vz* volume of distribution

### Efficacy

**Fig. 3:**
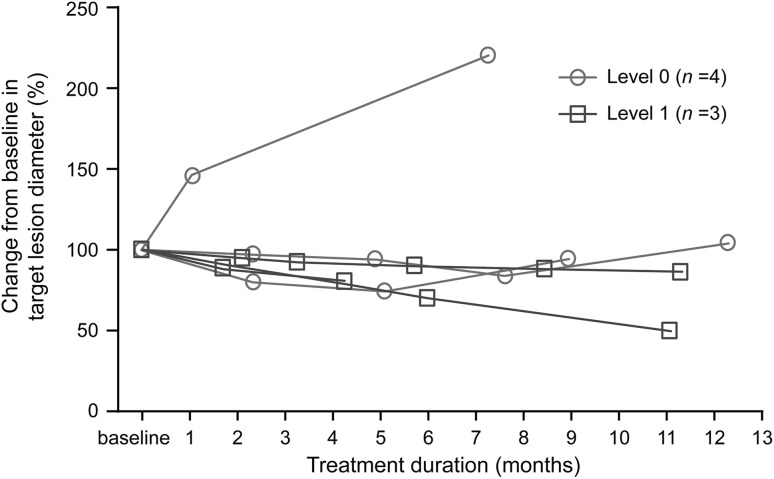
The time-course of changes in total diameters of the target lesion of individual patients. The data for one patient at level 0, who did not have baseline information on total diameters of the target lesion, are not shown on this figure

**Fig. 4:**
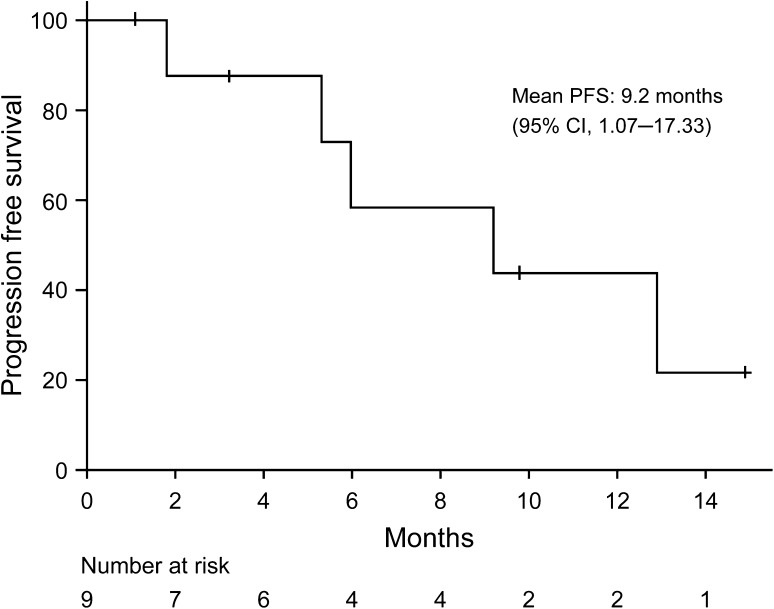
Kaplan–Meier curve for progression-free survival. *CI* confidence interval

Seven patients had measurable lesions, and were evaluated for efficacy. Although no patient achieved a partial response (PR) or complete response (CR) at level 0, three of four patients achieved stable disease (SD) and two of three patients had prolonged SD. Of three patients at level 1 (all with measurable disease), one patient achieved PR; the tumour shrank to 50% from baseline. Two patients achieved SD and one of the two patients had prolonged SD. The efficacy outcomes in terms of tumour response were considered equivocal; yet a response rate of 14.2% and a clinical benefit rate of 57.1% were estimated. The time-course changes in total diameters of the target lesion are presented in Fig. 3. OS was not assessed in the statistical analysis as no deaths were reported in this study. The estimated mean PFS (95% confidence interval) was 9.2 (1.07–17.33) months (Fig. 4).

## Discussion

We determined the safety and tolerability of eribulin in combination with capecitabine. Our results demonstrated that the dosing schedule of eribulin 1.4 mg/m^2^ on days 1 and 8 combined with capecitabine 1000 mg/m^2^ BID in a 2-weeks-on and 1-week-off schedule in a 21-day cycle was well tolerated and the toxicities were manageable in patients with MBC who were previously treated with anthracycline- or taxane-based regimens. Of all the three patients in this dosing schedule, two patients achieved clinical benefit (one PR and one long SD). Thus, we considered this dosing schedule suitable for further investigation in the phase II study.

In this study, the most common AE of ≥grade 3 was neutropenia, which is a common haematologic toxicity observed in patients treated with eribulin [7, 8, 18], whereas the incidence of neutropenia is lower with capecitabine-based chemotherapy compared with capecitabine-free chemotherapy [20]. The frequency of grade 3/4 neutropenia was 100% in our study as opposed to 66.7% in a previous study [16]. Although no patient experienced febrile neutropenia in this study, patients with MBC and their families should be cautioned about the neutropenic phase during treatment, given that the incidence of grade 3 or 4 neutropenia in eribulin monotherapy may be particularly high in East Asian patients, ranging from 85 to 95% [7, 21]. Other AEs such as hand–foot syndrome, anaemia, and fatigue were mild or moderate in severity and consistent with toxicities associated with capecitabine in pretreated patients with MBC [22, 23]. Incidences of AEs of special interest including hand–foot syndrome and peripheral neuropathy in our study were similar to a previous report [16]. Although the frequency of neutropenia was higher in our study, the overall incidences of AEs observed in this study were consistent with those in the previous study [16], indicating that ethnic differences did not affect the toxicity profiles of eribulin and capecitabine combination therapy. Grade 4 DLTs were experienced by one patient (increases in serum creatinine and uric acid); the pharmacokinetic data for this patient were similar to those of the other patients. The DLTs in this patient were considered to be tumour lysis syndrome, based on the syndrome criteria [24]. However, due to the early termination of the study, tumour shrinkage was not evaluated in this patient. Overall, the toxicity profile of eribulin and capecitabine observed in the present study is in line with previous reports on eribulin or capecitabine as monotherapy [8, 21–23, 25–27]. No new safety issues were reported.

We investigated the pharmacokinetics of eribulin in combination with capecitabine to assess the potential interaction between these drugs. According to our data, the plasma concentration and other pharmacokinetic parameters of eribulin were not influenced by co-administration of capecitabine when compared with previous studies on eribulin monotherapy in patients with MBC. Despite population-based differences, the results of the present study were consistent with the previous phase I dose-escalation study of eribulin in combination with capecitabine in patients with locally advanced or metastatic locally advanced cancer [15]. Additionally, the pharmacokinetic profiles of the combination of eribulin and capecitabine in our study were comparable to Caucasian patient population indicating that ethnicity did not influence the pharmacokinetic profiles in Japanese patients [28]. Capecitabine is sequentially metabolised via 5′-DFCR and 5′-DFUR to the active drug 5-FU by non-cytochrome-mediated reaction. The parameters of capecitabine and its metabolites in this study were consistent with the pharmacokinetic study in Japanese patients [29]. On the other hand, metabolism of eribulin is very limited and the drug is mainly excreted in faeces with more than 60% as the unchanged form [30]. No drug–drug interaction with CYP3A4 inducers/inhibitors on eribulin clearance was observed in humans, although CYP3A4 has a metabolic activity on eribulin [31, 32]. Thus, drug–drug interactions are not expected with eribulin and capecitabine. There is another oral fluoropyrimidine anticancer drug, S-1 that consists of tegafur, gimeracil, and oteracil potassium. Tegafur is a prodrug mainly metabolised by liver CYP2A6 to 5-FU. A phase I dose-escalation study of eribulin in combination with S-1 also demonstrated that the plasma concentration and other pharmacokinetic parameters of eribulin were not influenced by co-administration of S-1 [14]. Taken together, oral fluoropyrimidines, such as capecitabine and S-1, might not have pharmacokinetic drug interaction with eribulin.

We were unable to reach any conclusion regarding the antitumour activity of eribulin in combination with capecitabine because of the small number of patients and dose heterogeneity. Although the sample size was small, the mean PFS estimated in this study (9.2 ± 4.15 months) was numerically higher than the PFS estimated in the previous study by Twelves et al. [16]. The response rate of 14.2% (33% at level 1) observed in this study was lower than that of previous studies in which the combination of eribulin and capecitabine or S-1 was examined [14, 15]. In this regard, we could not find any specific differences in the baseline characteristics of patients in our study compared to previous studies that would explain the differences observed in the response rates. Nonetheless, the clinical benefit rate of 57.1% observed in our study indicated promising antitumour activity. Therefore, we believe that further investigation with a larger sample size at level 1 dosage would be important for conclusive estimation of the response rate in Japanese patients with MBC. In vitro and in vivo studies of TNBC cell lines demonstrated that the combination of eribulin and S-1 had synergistic antitumour effects [33]. In our study, there was one patient with TNBC who developed symptoms of tumour lysis syndrome following combination use of eribulin and capecitabine. Taken together, although we advise extreme caution on safety, a combination therapy of eribulin and capecitabine could show higher antitumor activity, especially for patients with triple-negative disease.

In conclusion, the MTD of the combination therapy of eribulin and capecitabine was not reached in this study, and the dosing schedule of eribulin 1.4 mg/m^2^ on days 1 and 8 combined with capecitabine 1000 mg/m^2^ BID in a 2-weeks-on and 1-week-off schedule in a 21-day cycle was considered to be suitable for further investigation in the phase II study. Further investigation of efficacy and safety of this combination regimen is warranted.

## Electronic supplementary material

Below is the link to the electronic supplementary material.

Supplementary material 1 (DOCX 28 kb)
