# Correction to: Phase I dose-finding study of eribulin and capecitabine for metastatic breast cancer: JBCRG-18 cape study

**DOI:** 10.1007/s12282-017-0815-7

**Published:** 2017-11-20

**Authors:** Masaya Hattori, Hiroshi Ishiguro, Norikazu Masuda, Akiyo Yoshimura, Shoichiro Ohtani, Hiroyuki Yasojima, Satoshi Morita, Shinji Ohno, Hiroji Iwata

**Affiliations:** 1 Department of Breast Oncology, Aichi Cancer Center, 1-1 Kanokoden, Chikusa-ku, Nagoya, 464-8681 Japan; 2 Department of Target Therapy Oncology, Graduate School of Medicine, Kyoto University, Kyoto, 606-8501 Japan; 3 Department of Surgery, Breast Oncology, NHO Osaka National Hospital, Osaka, 540-0006 Japan; 4 Division of Breast Surgery, Hiroshima City Hiroshima Citizens Hospital, Hiroshima, 730-8518 Japan; 5 Department of Biomedical Statistics and Bioinformatics, Graduate School of Medicine, Kyoto University, Kyoto, 606-8501 Japan; 6 Breast Oncology Center, Cancer Institute Hospital of JFCR, Tokyo, 135-8550 Japan

## Correction to: Breast Cancer https://doi.org/10.1007/s12282-017-0798-4

The article “Phase I dose-finding study of eribulin and capecitabine for metastatic breast cancer: JBCRG-18 cape study”, written by Masaya Hattori, Hiroshi Ishiguro, Norikazu Masuda, Akiyo Yoshimura, Shoichiro Ohtani, Hiroyuki Yasojima, Satoshi Morita, Shinji Ohno and Hiroji Iwata, was originally published electronically on the publisher’s Internet portal (currently SpringerLink) on 31 August 2017 without open access. With the author(s)’ decision to opt for Open Choice the copyright of the article changed on [9 November 2017] to © The Author(s) 2017 and the article is forthwith distributed under the terms of the Creative Commons Attribution 4.0 International License (http://creativecommons.org/licenses/by/4.0/), which permits use, duplication, adaptation, distribution and reproduction in any medium or format, as long as you give appropriate credit to the original author(s) and the source, provide a link to the Creative Commons license and indicate if changes were made.

The original article was corrected.

