# Supplementary material for: Phase I dose-finding study of eribulin and capecitabine for metastatic breast cancer: JBCRG-18 cape study
Source: Breast Cancer. 2017 Aug 31;25(1):108–17. doi: 10.1007/s12282-017-0798-4 (PMC5741785; doi:10.1007/s12282-017-0798-4)
Supplement: Supplementary file 1 — Supplementary material 1 (DOCX 28 kb) [file 12282_2017_798_MOESM1_ESM.docx]

Supplementary Table 1. Pharmacokinetic parameters of capecitabine and its metabolites

|  | | **Level 0 (N=6)** | | | | | | | | | | **Level 1 (N=3)** | | | | | | |
| --- | --- | --- | --- | --- | --- | --- | --- | --- | --- | --- | --- | --- | --- | --- | --- | --- | --- | --- |
|  | **5’-DFCR** | | | **5’-DFUR** | | | **5-FU** | | | **5’-DFCR** | | | | **5’-DFUR** | | **5-FU** | |  |
|  | Day 0 | | Day 1 | | Day 0 | Day 1 | | Day 0 | Day 1 | | Day 0 | | Day 1 | Day 0 | Day 1 | Day 0 | Day 1 |  |
| C_max_, µg/mL |  | |  | |  |  | |  |  | |  | |  |  |  |  |  |  |
| mean | 3.343 | | 3.075 | | 4.897 | 4.521 | | 0.111 | 0.137 | | 4.032 | | 3.231 | 4.136 | 5.260 | 0.149 | 0.195 |  |
| SD | 1.630 | | 1.520 | | 1.775 | 2.150 | | 0.043 | 0.075 | | 1.878 | | 1.574 | 1.876 | 4.028 | 0.077 | 0.136 |  |
| AUC_0-6h_, h·µg/mL |  | |  | |  |  | |  |  | |  | |  |  |  |  |  |  |
| mean | 6.928 | | 6.876 | | 9.425 | 9.628 | | 0.215 | 0.269 | | 10.049 | | 6.002 | 10.140 | 8.361 | 0.351 | 0.343 |  |
| SD | 2.594 | | 3.614 | | 2.177 | 4.748 | | 0.054 | 0.142 | | 2.336 | | 1.398 | 1.737 | 4.650 | 0.088 | 0.191 |  |
| t_1/2_, h |  | |  | |  |  | |  |  | |  | |  |  |  |  |  |  |
| mean | 0.8 | | 0.8 | | 0.7 | 0.7 | | 0.7 | 0.7 | | 0.7 | | 0.7 | 0.7 | 0.6 | 0.8 | 0.5 |  |
| SD | 0.2 | | 0.2 | | 0.2 | 0.2 | | 0.3 | 0.2 | | 0.1 | | 0.1 | 0.1 | 0.1 | 0.2 | 0.1 |  |
| MRT, h |  | |  | |  |  | |  |  | |  | |  |  |  |  |  |  |
| mean | 1.9 | | 1.6 | | 1.8 | 1.5 | | 1.8 | 1.4 | | 1.9 | | 1.5 | 1.9 | 1.5 | 1.9 | 1.6 |  |
| SD | 1.2 | | 0.7 | | 1.3 | 0.7 | | 1.3 | 0.8 | | 0.1 | | 1.5 | 0.2 | 1.7 | 0.1 | 1.6 |  |

AUC_0-6h_, area under the area under the plasma concentration-time from 0h to 6h; C_max_, maximum plasma concentration; CL, clearance; MRT, mean residence time; SD, standard deviation; t_1/2_, half-life; Vz, volume of distribution.
